# Meta-analysis of nasopharyngeal carcinoma microarray data explores mechanism of EBV-regulated neoplastic transformation

**DOI:** 10.1186/1471-2164-9-322

**Published:** 2008-07-07

**Authors:** Xia Chen, Shuang Liang, WenLing Zheng, ZhiJun Liao, Tao Shang, WenLi Ma

**Affiliations:** 1 Institute of Genetic Engineering, Southern Medical University, Guangzhou, PR China; 2 Xiangya Pingkuang associated hospital, Pingxiang, Jiangxi, PR China; 3 Southern Genomics Research Center, Guangzhou, Guangdong, PR China

## Abstract

**Background:**

Epstein-Barr virus (EBV) presumably plays an important role in the pathogenesis of nasopharyngeal carcinoma (NPC), but the molecular mechanism of EBV-dependent neoplastic transformation is not well understood. The combination of bioinformatics with evidences from biological experiments paved a new way to gain more insights into the molecular mechanism of cancer.

**Results:**

We profiled gene expression using a meta-analysis approach. Two sets of meta-genes were obtained. Meta-A genes were identified by finding those commonly activated/deactivated upon EBV infection/reactivation. These genes could be key players for pathways de-regulated by EBV during latent infection and lytic proliferation. Meta-B genes were obtained from differential genes commonly expressed in NPC and PEL (primary effusion lymphoma). We then integrated meta-A, meta-B and associated factors into an interaction network using acquired information. Our analysis suggests that NPC transformation depends on timely regulation of DEK, CDK inhibitor(s), p53, RB and several transcriptional cascades, interconnected by E2F, AP-1, NF-κB, STAT3 among others during latent and lytic cycles.

**Conclusion:**

In conclusion, our meta-analysis strategy re-analyzed EBV-related tumor data sets and identified sets of meta-genes possibly involved in maintaining latent or switching to lytic cycles of EBV in NPC. The results of this analysis may shed new lights to further our understanding of the EBV-led neoplastic transformation.

## Background

Nasopharyngeal carcinoma (NPC), whose onset can be found in the epithelial cells of the nasopharyngeal region, causes a high incidence of fatality in patients mostly in southern China and southeast Asia [1]. Epstein-Barr virus (EBV), a ubiquitous human herpes virus, is thought to be closely associated with NPC, as well as other hematopoietic malignancies such as African Burkitt's lymphoma, primary effusion lymphoma (PEL), Hodgkin's disease, and adult T-cell leukemia. Although infection by EBV occurs in most individuals, it is usually asymptomatic. EBV is orally transmitted and can be detected in oropharyngeal secretions from infected individuals [2]. Subsequently EBV settles in resting B lymphocytes and renders infected B cells immortalized and unrestricted for proliferation [3]. Some lines of evidence suggest that EBV enters B cells by pairing its glycoprotein gp350/220 with the complement receptor (CR2/CD21) [4]. Once in the primarily infected host, this virus can establish a long and persistent latent infection during which only few viral genes are active, presumably to escape cellular defense. Several viral proteins including EBNA1, LMP1 and LMP2 are active to maintain and regulate this latent state. The lytic production occurs after a long viral latency and can be triggered by spontaneous or artificially-induced reactivations, and eventually leads to the production of a large number of virions released through cell lysis. This is accompanied by the expression of certain lytic genes. Z protein, encoded by viral *BZLF1 *gene, is a potent transactivator of multiple viral and cellular genes critical for switching from latent to lytic cycle. Epithelial cells generally do not express CD21 *in vivo *and can be infected *in vitro *by direct contact with virus-containing cells or supernatant. This suggests that epithelial tissues might be infected by being close to lytically infected B cells. It remains to be shown that the transforming potential of EBV might ultimately contribute to the pathogenesis of NPC.

Currently, NPC studies aim to achieve the following objectives: providing an early and sensitive diagnosis, and trying to understand the molecular basis underlying the disease formation [5,6]. The availability of the human genome sequence, a large collection of microarray expression data together with the development of bioinformatics will enable us to achieve these objectives. The Gene Expression Omnibus (GEO) [7] has made available hundreds of thousands of experimental data of gene expression for users to explore. However, the interrelationship of many these data sets has not been explored. To identify genes associated with various cancers, techniques such as filtering by fold change, expression level or significance flag, as well as statistical analysis (for instance *t*-test and ANOVA) have been applied to select candidate genes associated with tumorigenesis [8,9]. With these simple screening techniques for a given data set, one might end up with hundreds if not thousands of genes needed for further validation. Recently, research exploring interactions and regulatory networks of selected genes and their products began to gain momentum in studying diseases [10,11]. Many computational methods have been developed to facilitate expression data analysis. Gene clustering, pathway analysis and gene ontology (GO) analysis are commonly used [12-14]. Moreover, literature mining enables us to extract the meaningful biological information from publications and to identify known networks or pathways [15,16]. The information, collected from human curation and comprehension of specific experiments, is very important in our analysis to further our understanding of the etiology of NPC.

In this study, we have utilized a meta-analysis approach to identify meta-genes across different data sets. This is based on the belief that those significant genes shared by multiple data sets could be the ones which are more important to focus on. This allows us to turn our attention and resources to potentially high value targets as they are less likely to be derived from randomness of analysis. Using such strategy, we have identified two sets of meta-genes (meta-A and meta-B) and discussed the potential roles some of them might play in the course of EBV-related neoplastic transformation.

## Results

### Screening strategy for meta-genes

**Figure 1:**
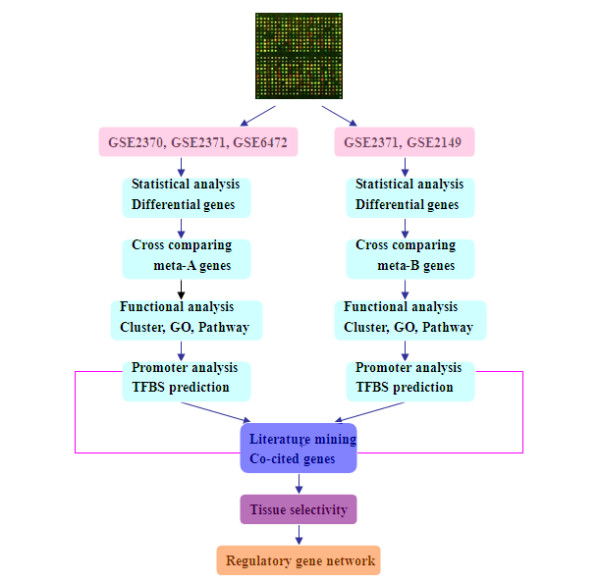
**The workflow of our strategy**. The red lines represent the iteration between TFBSs prediction and literature mining.

To overcome the weakness of conventional microarray-based data analysis, meta-analysis was applied to heterogeneous microarray data of various origins [11,17]. We designed a strategy (the workflow is shown in Figure 1) to build up lists of meta-genes in EBV-positive tumors. This can be organized in two phases. In phase one, we first analyzed data sets derived from EBV primary infection and lytic production to identify meta-genes de-regulated by EBV when switching to lytic cycle. Next, we extracted differential genes shared by two EBV^+^tumors (NPC and PEL) to find meta-genes commonly de-regulated by EBV. In phase two, gene clustering, pathway and network prediction were done in four steps: *(i) *Meta-genes were classified based on known functional categories and similar ontological terms; *(ii) *Over-represented transcription factor binding sites (TFBSs) were predicted; *(iii) *Literature mining was conducted to analyze transcription factors that are co-cited with the meta-genes and *(iv)*, Tissue specificity and subcellular localization of the meta-genes were analyzed. Finally, we integrated all the above information into a gene interaction network and proposed our hypothesis.

### Differential genes

**Figure 2:**
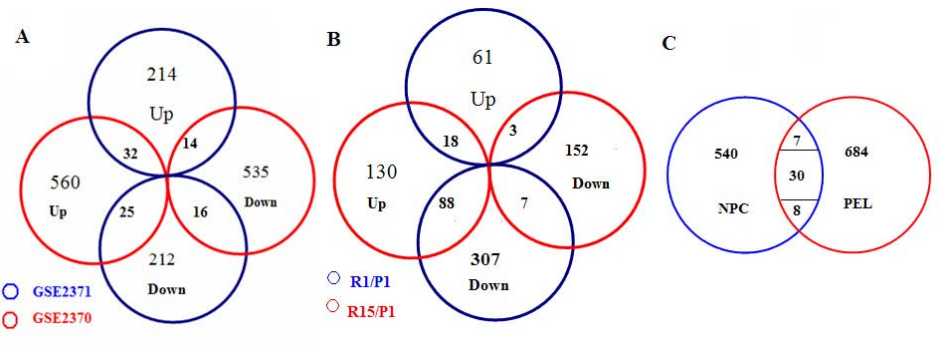
**Venn diagrams of the differential genes identified from the data sets used**. (A) Intersection of differential genes between GSE2370 and GSE2371; (B) Intersection of differential genes between R15/P1 and R1/P1 of GSE6472; (C) Intersection of differential genes between GSE2371 and GSE2149.

The Venn diagram in Figure 2A shows the distribution of differential genes between GSE2370 (EBV^-^/normal) and GSE2371 (EBV^+^/EBV^-^). In brief, of the 260 differentially up-regulated genes in GSE2371, 32 were also up-regulated and 14 others were found down-regulated in GSE2370. Of the 253 genes in the down-regulated group in GSE2371, 25 genes were up-regulated and 16 were down-regulated in GSE2370. A total of 87 differential genes were identified as likely targets by EBV during primary infection. Many of these genes have been discussed [18].

Figure 2B shows the Venn diagram of differentially expressed genes between primary infection and reactivation in GSE6472. Of the 82 differentially up-regulated genes in R1 (initial reactivation), 18 genes were up-regulated and 3 were down-regulated in R15 (recurrent reactivation). Of the 402 genes down-regulated in R1, 88 genes were up-regulated and 7 were down-regulated in R15. A total of 116 differential genes were found in common between R1 and R15.

When cross-comparing these 116 differential genes expressed during EBV reactivation to the 87 differential genes found during primary infection, 23 meta-genes (named as meta-A, Table 1) were found to be the key candidates responsive to EBV.

**Table 1:** List of 23 meta-A genes between the EBV-reactivation and EBV^+^/EBV^-^-NPC

Latent infection expression	Recurrent infection expression	Gene symbol	Total
Up-regulated	Up-regulated	***MAP3K5, TOP1, EMP3, GNG7***	4
Up-regulated	Down-regulated	***FCGBP1, KMO, PSPH, PITX1, DEK, RPS28***	6
Down-regulated	Up-regulated	***ITGA6, PPP2R2D, SMARCC1***	3
Down-regulated	Down-regulated	***DUSP1, ST5, APPBP1, DUSP6, TRIP12, PABPC1, TKT, CD9, IMPDH2, HOXA9***	10

The 585 differential genes in GSE2371 (EBV^+^-NPC) and 729 genes in GSE2149 (EBV^+^-PEL) were integrated in Figure 2C. The intersection represents 45 overlapping meta-genes (named as meta-B, Table 2) expressed in both tumor types, including a group of 30 common genes (20 commonly up-regulated and 10 commonly down-regulated in both NPC and PEL), 7 up-regulated in NPC but down-regulated in PEL, and 8 down-regulated in NPC but up-regulated in PEL. It is interesting to note that meta-A genes and meta-B genes, also referred to meta-genes collectively, share three genes in common: *DEK, DUSP1 *and *ITGA6*.

**Table 2:** List of 45 meta-B genes between EBV^+^/EBV^-^-NPC and EBV^+^/EBV^-^-PEL

Expression in NPC	Expression in PEL	Gene symbol	Total
Up-regulated	Up-regulated	***BMP1, BTG1, CAV1, CAV2, CD53, DEK, EFEMP1, GADD45A, GALNT3, GAS7, GATM, ITGA6, LLGL2, LSP1, SEC14L1, UBE1L, INPP1, PGRMC1, PHGDH, TGIF***	20
Down-regulated	Down-regulated	***CTSS, EIF5A, FHL2, INSIG1, LYN, MME, OAS1, PYGL, TAF15, ALDH2***	10
Up-regulated	Down-regulated	***CDKN1A, LAMC1, LY6E, MFAP2, RB1, RPL10, SQSTM1***	7
Down-regulated	Up-regulated	***DUSP1, JUNB, KRT5, MGST3, SEPP1, SPTBN1, ITGAV, NFKBIA***	8

### Functional analysis and gene annotation

23 meta-A genes listed in Table 1 are mainly involved in MAPK signal cascade (*p *= 0.047), macromolecule metabolism (*p *= 0.021), phosphorylation (*p *= 0.037), biopolymer metabolism (*p *= 0.008), protein complex (*p *= 0.028), cellular metabolism (*p *= 0.042) and organ morphogenesis (*p *= 0.037) based on DAVID (Database for annotation, visualization and integrated discovery) analysis. The 45 meta-B genes in NPC and PEL are related to organelle lumen (*p *= 0.044), cellular physiological process (*p *= 0.030), macromolecule metabolism (*p *= 0.050), ribonucleoprotein (*p *= 0.038), regulation of cell process (*p *= 0.048), cell adhesion (*p *= 0.012) and transferase activity (*p *= 0.018).

### TFBSs prediction

TELiS analysis (*p *< 0.05) revealed that HLF-01, ATF-01, MYCMAT-01, E2F-01, CREB-02, NFE2-01, MAX-01, CREB-01, TATA-01 and OCT-01 are over-represented within the proximal promoter region of many meta-A genes. We then looked for any common regulatory module by sifting through each of the promoter sequences. As a result, *DUSP1, IMPDH2, RPS28, TOP1, PBPC1 *and *EMP3 *found in our study share these two TFBSs: ATF and CREB.

The results of the Genomatix Bibliosphere analysis showed that *DEK, PITX1, TGIF1, RB *and *JUNB *encode for transcription factors/activators. Transcription factor RB is known to bind E2F; TGIF can complex with TALE; JUNB associates with AP1F. Moreover, RB was often co-cited with DEK, CDKN1A and GADD45A [19].

### Tissue specificity and subcellular localization

Lymph node, one reservoir of resting B cells latently infected by EBV after primary infection, was chosen as a closely related tissue for NPC because of the absence of nasopharyngeal epithelia data when studying tissue specificity. Previous study has generated a list of tissue selective genes among which 34 are highly expressed in lymph node [20]. When comparing genes found in this study (prior to cross-comparison) with the 34 genes (please see the Additional file 1), no intersection was found.

Analysis using GeneCards showed that most meta-genes and related transcription factors expressed predominantly in blood tissue. *CD9, ITGA6, CDKN1A, TP53, EGR1 *and *ST5 *have been reported to be related to many tumor types including squamous epithelium tumor. In addition, most differential genes are localized either to nucleus or cytoplasm, except that *CD9 *and *ITGA6 *encode for membrane proteins. *CDKN1A, RB, DEK, Daxx *and *MAP3K5 *genes, which are downstream of the *BZLF1 *pathway, all reside on chromosome 6.

### Regulatory network

**Figure 3:**
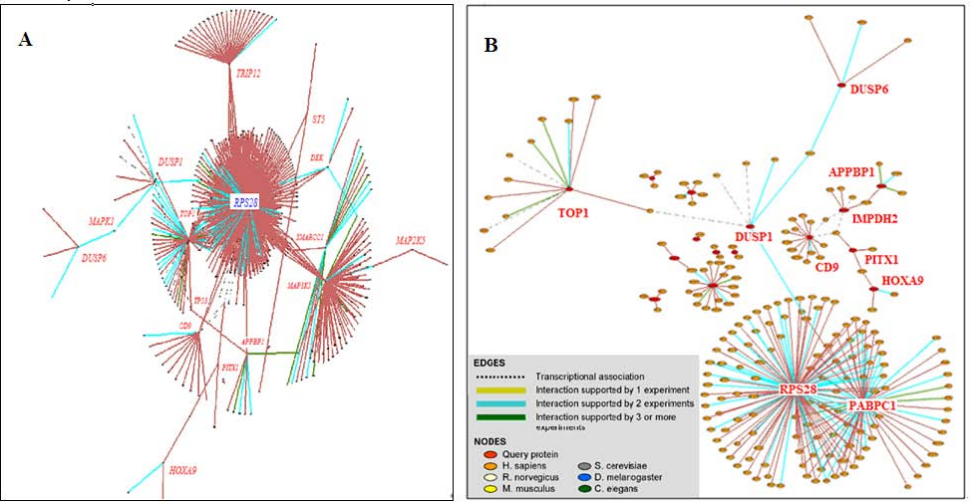
**Visualization of physical interactions and transcriptional associations of meta-A genes**. (A) Global network involving the 23 meta-A genes labeled in red with the extended subnets centered on interaction input. (B) Close-up of two subnets of many input genes. For better viewing experience and more details, please see the Additional file 2.

23 meta-A genes were used as input into pSTIING to visualize any known functional associations, physical interactions or transcriptional regulations (Figure 3A, global view; Figure 3B, close-up; see Additional file 2). There exist two main subnets: one contains *APPBP1, CD9, PITX1 *and *SP1*, the other one involves *DUSP1, TOP1, RPS28 *and *PABPC1*.

**Figure 4:**
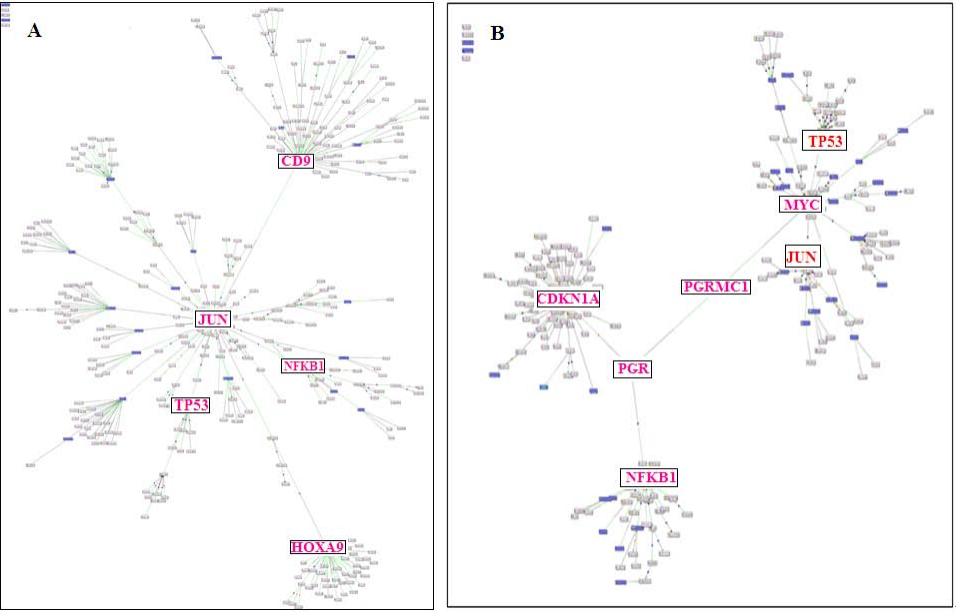
**Network of meta-A genes or meta-B genes by adding the related transcriptional factors**. (A) Network of the 23 meta-A genes in EBV infected cells linked by some related transcriptional factors. The main nodes involves transcription factors JUN, CD9 and HOXA9. (B) The network of the 45 meta-B genes connected by a few related transcriptional factors: CDKN1A, NFKB1 and MYC. Readers are referred to the Additional file 3 for more details.

Literature mining using iHOP was conducted to find support for the proposed networks. Based on existing knowledge, a few more related transcription factors such as JUN, MYC, PGR and NFKB1 were added by Genomatix BiblioSphere to connect the 23 meta-A genes (Figure 4A) or the 45 meta-B genes (Figure 4B). As shown in Figure 4B, most of the 45 meta-B genes cluster around CDKN1A, RB, JUN, NFKB1, TP53 and MYC (see Additional file 3).

**Figure 5:**
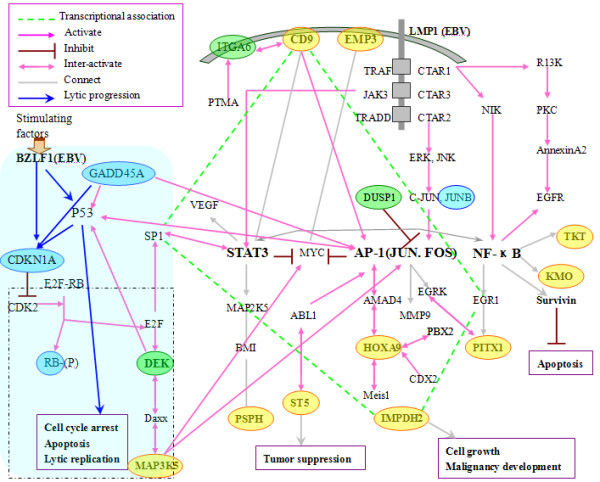
**Regulatory network of important genes involved in EBV-dependent NPC**. The genes circled in yellow represent the meta-A genes involved in different life cycles of EBV. Those circled in blue represent the meta-B genes found in EBV^+^-tumors. Those circled in green are three genes in common between meta-A genes and meta-B genes. The area shadowed in gray on the lower left shows the *BZLF1 *pathway in latent and lytic replication. The dotted rectangle shows the common DEK/E2F pathway. The genes, framed in rectangle, were up-regulated during latency while down-regulated in lytic proliferation. And the green dashed lines represent the circuit of the transcriptional association of *SP1, IMPDH2, CD9 *and *EGR1*.

Having integrated all the above information, we obtained a regulatory network of our meta-genes found to be related to NPC (Figure 5).

## Discussion

Four microarray data sets (Table 3) were chosen to explore the molecular mechanism of EBV-dependent NPC in our study. A few points can be drawn from this study as follows. *(i) *EBV seems to have a preference of targeting differentially expressed genes than those expressed ubiquitously in NPC cells [18]. This suggests that infecting EBV triggers cellular changes by de-regulating many factors in signal transduction or regulatory pathways in order to remain in its host after primary infection. *(ii) *Nonetheless, only a fraction of these genes (meta-A genes) stay differential during recurrent EBV reactivations and most other genes return to stable expression gradually. Those remain differentially expressed (about a quarter of the original number) during recurrent reactivation worth more attention. They might be responsible for subsequent cellular transformation and possibly metastasis by spreading the virions through EBV's lytic proliferation and transforming more vulnerable host cells into NPC. *(iii) *The 45 meta-B genes shared by EBV-associated NPC and PEL would give important clue to understand the common pathogenesis of the EBV-led pathogenesis. The fact that both meta-A and meta-B gene sets share *DEK, DUSP1 *and *ITGA6 *in common indicates that all three cancer-related genes are more important to look at among all others.

**Table 3:** List of the data sets used in this research

Accession number	Gene chips' type	Samples (cell lines)
GSE2370	7500 K microarray	NPC(TW01, TW03, TW04, TW06, CGBM1)/normal nasal nucosal epithelia
GSE2371	7500 K microarray	EBV^+^/EBV^-^-NPC(TW01, TW03, TW04, TW06, CGBM1)/common reference RNAs
GSE6472	Agilent 4410B	EBV reactivations in NPC (P1/P15/R1/R15)
GSE2149*	Affymetrix HG-133A	EBV^+^/EBV^-^-PEL

* Nine cell lines (BC-1, BC-2, BC-3, BC-5, BCBL-1, BCKN, IBL-4, PEL-5 and SM1) derived from patients with lymphomatous effusions and three PEL patient samples were used in GSE2149.

With knowledge gathered by in-depth analysis, a detailed regulatory network was set up by joining newly identified meta-genes with related transcriptional factors. As shown in Figure 5, many of our meta-genes are involved in pathways rooted by LMP1 and *BZLF1*. A transcriptional circuit involving *SP1*, *CD9*, *EGR1 *and *IMPDH2 *connects three pathways led by LMP1 to the *BZLF1 *cascade through the inter-network between *SP1 *and *STAT3 *[21]. It is worth noting that E2F binding site can be found within the promoter region of SP1 [22], and SP1 binding site can be found within the EBV early promoter [23,24]. This suggests that SP1 may be one of the key players in switching between the latent infection and lytic proliferation. The associations among meta-genes suggest that EBV latent infection probably depends on important regulators such as JUN, MYC, NF-κB, and p53 as previous thought [25,26].

In latent infection, CDK2 activity is needed to maintain cell cycle progression and to phosphorylate RB. The pairing of RB/E2F as a complex plays important role in cell cycle regulation, apoptosis, differentiation [27] and EBV replication [28]. When RB gets hyperphosphorylated, E2F is released from the complex to transactivate its target genes needed for proliferation. In line with our prediction, expression of *DEK *has been shown to be targeted and activated directly by E2F [29]. DEK, an abundant and ubiquitous chromatin protein and transcription repressor [30], can then regulate JUN, MYC, and p53 through Daxx and MAP3K5. For example, DEK can inhibit apoptosis by interfering with p53 [31]. It has also been reported that RB-dependent over-expression of DEK blocks senescence or apoptosis of infected cells [31,32]. Cell death in response to *DEK *knockdown was accompanied by increased protein stability and transcriptional activity of the p53 tumor suppressor [31]. When RB loses its activity, expression of both E2F and DEK becomes up-regulated [33].

*BZLF1 *and *BRLF1*, the switches from latency to lytic infection, are the drivers of the EBV lytic replication [34]. Their expression are inactive in latent cells but can be activated by a number of triggers [35-37]. The activation depends on the existence of specific binding sites in their promoters, some of these binding sites can be bound by SP1, CREB, ATF-1/2 and c-JUN [38,39]. We predicted that the forming of ATF/CREB heterodimers, also commonly found in Hodgkin's disease [40], may be important for regulating *BZLF1 *during recurrent reactivation. Expression of the Z protein, encoded by *BZLF1*, is known to arrest cell cycle progression in several epithelial tumor cell lines lacking the entire EBV genome. Such arrest is mediated by Z-induced expression of p53 and two inhibitors of CDK, namely p21 (CDKN1A/CIP-1) and p27 (KIP-1), followed by the accumulation of the underphosphorylated RB protein and the down-regulation of EBV immediate-early and early proteins [41].

Expression level of *DEK *is much lower in reactivation state than in latent state. The lack of E2F released from the hypophosphorylated RB-E2F complex may have a causal effect on the down-regulation of DEK and thus promotes apoptosis in the presence of apoptotic factors such as p53. This suggests that DEK may have been down-regulated in response to *BZLF1 *activation to favor the lytic cycle. Comparing to latent cycle, the lytic cycle produces infectious virions up to 1000 folds and possibly leads to the infection and transformation of more host cells. The accumulative effect of this could ultimately leads to aggressive tumor growth and metastasis. The potent lytic inducer *BZLF1 *has been explored to treat EBV^+ ^tumors [42,43]. *BZLF1*, if over-expressed exclusively in tumor cells using a tumor-specific vector (such as a specially-designed adenoviral vector), could induce potent cell lysis and serve as a general strategy to treat many cancers.

Our meta-analysis approach re-analyzed four EBV-related tumor data sets and identified meta-genes using expression profiling and integrated bioinformatics. Based on this information, we constructed a gene network to better our understanding of EBV-regulated neoplastic transformation. It should be pointed out that we have not specifically addressed the false discovery rate directly and thus our statistical analysis might have unavoidably produced some false positive hits or missed some important genes. However, gene set intersection can somehow prevent a large number of random genes from entering into our selection. Like any other analytical approach, this process depends on data quality and completeness. It may not identify all the desirable inner networks if data is sub-optimal.

## Conclusion

This study has identified two sets of meta-genes, including 23 meta-A genes expressed differentially when switching to recurrent reactivation, and 45 meta-B genes expressed in both EBV-dependent NPC and PEL. The integrated meta-gene network suggests that NPC transformation is likely to depend on timely regulation of DEK, CDK inhibitor(s), p53, RB and several transcriptional cascades, interconnected by E2F, AP-1, NF-κB, STAT3 among others during EBV's life cycle. The result of this analysis demands for further investigation to validate and to justify. More data analyses are needed to support and to complement ours in order to explore thoroughly the molecular mechanism of NPC. It is hope that research like this could point to the right direction for conquering this deadly disease eventually. In the meanwhile, the causal effect of EBV for NPC remains for open discussion even though it is known for long that EBV is omnipresent in NPC. Future research should also pay attention to impacts of other factors as well since NPC is quite restricted to some local populations and geographic locations. These factors include environmental, dietary ones in addition to ethnic genetic susceptibility and polymorphism.

## Methods

### Data sets

Four data sets retrieved from the GEO database are listed in Table 3 and the open-access analysis tools selected are shown in Table 4. Data sets GSE2370 [44] and GSE2371 [45] submitted by Lee contain 15 samples surveyed by the 7500 K microarray representing approximately 7411 distinct human transcripts expressed in five representative NPC cell lines: TW01, TW03, TW04, TW06 and CGBM1. TW01 is a *Homo sapiens *NPC cell line derived from a keratinizing squamous cell carcinoma; TW03 is derived from a lympho-epitheliomatous undifferentiated NPC; TW04 and TW06 are derived from two distinct undifferentiated carcinomas; and CGBM1 line is derived from bone marrow metastatic NPC tumor tissue. GSE 2370 used the five EBV^- ^NPC cell lines (labeled with cy5) mentioned above against normal nasal mucosal epithelial (labeled with cy3) as a control. GSE2371 used the same five EBV^- ^NPC cell lines and EBV^+ ^cell lines (labeled with cy5) against common reference RNAs (labeled with cy3). Dataset GSE6472, supplied by Chia [46] and based on Agilent 4410B microarry, contains three groups of expression data (R1/P1, R15/P1 and P15/P1) representing different EBV reactivations of NPC-TW01 cell line using dye-swap. P1, an EBV-positive NPC cell line (NA) derived from NPC-TW01 infected with recombinant Akata EBV but without having EBV reactivation, serves as the source of primary reference sample. P15 refers to latently infected NA cell line subjected to 15 times or more regular passages of EBV without having EBV reactivation. R1 and R15 are NA cells experienced EBV recurrent reactivation one and fifteen times induced artificially by sodium *n*-butyrate (SB) and 12-*o*-tetradecanoylphorbol-13-acetate (TPA), respectively. Dataset GSE2149, supplied by Fan [47] and based on the Affymetrix HG-133A microarray, has eleven samples (21 microarrays) from EBV^+^/EBV^-^-PEL. More information of the four data sets is shown in Table 3.

**Table 4:** Web resources used

Name	Address	Content used
GEO		Accession numbers GSE2370, GSE2371, GSE2149 and GSE6472, Microarray data
Genomatix		BiblioSphere, Matlinspector, literature mining
iHOP		Literature mining
DAVID		Pathway and GO classification
GO		GO terms, biological process, molecular function and cellular component
TELiS		TFBSs information
pSTIING		Networks of gene interactions
GeneCards		Subcellular localization and tissue specificity

### Data preprocessing

The raw data from each experiment was normalized using Lowess smoother (per spot and per chip: intensity-dependent normalization) for data sets GSE2370, GSE2371 and GSE6472, or using median over entire array for GSE2149 to minimize randomness of signals among microarrays and spots. To focus on high-quality and stronger hybrid signal spots, we excluded all data points whose signal intensities below 100. Filtering on flags, which we required all present calls only, was applied to GSE2370 and GSE2371. Filtering on expression level with threshold of standard error average× 4 were used for GSE6472. Probes with 20% data points missing were then filtered out for GSE2149.

### Selection of differential genes

We utilized GeneSpring GX 7.3.1 (Agilent technologies, US) to analyze two-channel data and BRB ArrayTools 3.5.0 (Dr. Richard Simon and Amy Peng Lam) to analyze one-channel data. GeneSpring GX was used to analyze GSE2370, GSE2371 and GSE6472 using cross gene error model [48]. The following thresholds were used to obtain sets of differential genes as close to those described by the authors of the data sets as possible. The statistical comparison (*p *< 0.05) of GSE2370 revealed that 1182 genes were differentially expressed, including 617 genes with greater than 1.765 fold-changes as an up-regulated group and 565 genes with less than -1.765-fold defined as a down-regulated group. Similarly, analysis of GSE2371 revealed that 513 were differentially expressed, including 260 genes showing greater than 1.25-fold as up-regulated group and 253 showing less than -1.25-fold as down-regulated group. The differential genes identified from analyzing GSE2370 and GSE2371 were designated as potential target genes of primary EBV infection.

Up-regulated or down-regulated genes in GSE6472 were identified using an absolute threshold of 1.5-fold. Then, the differential genes of R1/P1/R15/P15 were cross-compared to those from GSE2371 to obtain meta-A genes which are targeted by EBV and subjected to EBV reactivation of various duration and frequency.

GSE2371 and GSE2149 come from EBV^+^/EBV^-^-NPC and EBV^+^/EBV^-^-PEL respectively. We collected the common differential meta-B genes infected by EBV between the two tumors by cross-comparing the gene sets obtained after analyzing the two data sets using BRB ArrayTools. Genes showing an absolute 1.5 fold-changes (*p *< 0.05) in either direction were counted as either up-regulated or down-regulated.

### Functional analysis and gene annotation

We postulate that the differentially expressed genes we identified may be functionally related and not independent. Hierarchical clustering and K-means clustering [13,49], two popular methods to infer similar regulation or biological function, were used to create gene clusters based on similar expression patterns. DAVID (NIAID, NIH, USA) [50], a functional annotation tool, was used to analyze the enriched metabolic and signal pathways, as well as GO terms of biological process (BP), molecular function (BF), and cellular component (CC).

### TFBSs prediction

The differentially expressed genes related to NPC, which is a complicated disease, might be co-regulated by a regulatory module rather than any individual factor. Therefore, we searched for TFBSs using Transcription Element Listening System (TELiS) (Weihong Yan, Steve Cole, USA) [51] with a default of 600 bp upstream within the transcription start site and a filtering stringency of 90%. TFBSs prediction was also done with Genomatix's Matlinspector (Munich, Germany) accompanied by literature mining to confirm the correlation of the involved transcription factors.

### Integration and construction of a regulatory network

iHOP [52] was used to conduct literature-mining to uncover significant pairs among the differential genes. Regulatory networks which represent gene interactions correlated with transcription profiling were modeled by the Genomatix's Bibliosphere software. pSTIING, which stands for protein, signaling, transcriptional interactions and inflammation networks gateway [53], was used to describe and to confirm the known interactions and transcriptional associations of these differential genes. The regulatory network in NPC with EBV infection was constructed based on the acquired knowledge.

### Tissue Selectivity

Tissue-specific/selective gene expression is believed to be of physiological importance [54]. We compared our genes with those found to be tissue-selective from previous analysis of the BioExpress database [20]. Lymph node and nasopharyngeal epithelia data were considered to be two important tissues for EBV infection even though the mechanism for EBV entry into epithelial cells and maintenance of latency is less well understood. In the absence of nasopharyngeal epithelia-selective genes, we opted to compare our meta-genes with those found to be lymph node-selective. Subcellular localizations of our genes and their products were identified using GeneCards [55,56] to complement the regulatory network.

## Authors' contributions

XC and SL conceived, designed the meta-analysis and drafted the manuscript. XC carried out the bioinformatics analysis. ZJL and TS assisted in analytic tools. WLM and WLZ initiated the project and supervised the graduate program. All authors read and approved the manuscript.

## Supplementary Material

Additional file 1 **34 lymph node-selective genes**. This file shows the gene IDs, gene names and gene symbols of 34 lymph node-selective genes. Click here for file

Additional file 2 Figure 3. This file contains two figures of visualization of physical interactions and transcriptional associations of meta-A genes. (A) Global network involving the 23 meta-A genes labeled in red with the extended subnets centered on interaction input. (B) Close-up of two subnets of many input genes. Click here for file

Additional file 3 Figure 4. This file contains two figures of the network of meta-A genes or meta-B genes by adding the related transcriptional factors. (A) Network of the 23 meta-A genes in EBV infected cells linked by some related transcriptional factors. The main nodes involves transcription factors JUN, CD9 and HOXA9. (B) The network of the 45 NPC-PEL meta-B genes connected by a few related transcriptional factors: CDKN1A, NFKB1 and MYC. Click here for file
